# Maize yield prediction with trait-missing data via bipartite graph neural network

**DOI:** 10.3389/fpls.2024.1433552

**Published:** 2024-10-04

**Authors:** Kaiyi Wang, Yanyun Han, Yuqing Zhang, Yong Zhang, Shufeng Wang, Feng Yang, Chunqing Liu, Dongfeng Zhang, Tiangang Lu, Like Zhang, Zhongqiang Liu

**Affiliations:** ^1^ Information Technology Research Center, Beijing Academy of Agriculture and Forestry Sciences, Beijing, China; ^2^ National Innovation Center for Digital Seed Industry, Beijing, China; ^3^ Beijing Key Laboratory of Multimedia and Intelligent Software Technology, Beijing Institute of Artificial Intelligence, Beijing University of Technology, Beijing, China; ^4^ National Agro-Tech Extension and Service Center, Beijing, China; ^5^ Beijing Digital Agriculture Promotion Center, Beijing Municipal Bureau of Agriculture and Rural Affairs, Beijing, China

**Keywords:** yield prediction, graph neural network, bipartite graph, data imputation, gradient harmonization

## Abstract

The timely and accurate prediction of maize (*Zea mays* L.) yields prior to harvest is critical for food security and agricultural policy development. Currently, many researchers are using machine learning and deep learning to predict maize yields in specific regions with high accuracy. However, existing methods typically have two limitations. One is that they ignore the extensive correlation in maize planting data, such as the association of maize yields between adjacent planting locations and the combined effect of meteorological features and maize traits on maize yields. The other issue is that the performance of existing models may suffer significantly when some data in maize planting records is missing, or the samples are unbalanced. Therefore, this paper proposes an end-to-end bipartite graph neural network-based model for trait data imputation and yield prediction. The maize planting data is initially converted to a bipartite graph data structure. Then, a yield prediction model based on a bipartite graph neural network is developed to impute missing trait data and predict maize yield. This model can mine correlations between different samples of data, correlations between different meteorological features and traits, and correlations between different traits. Finally, to address the issue of unbalanced sample size at each planting location, we propose a loss function based on the gradient balancing mechanism that effectively reduces the impact of data imbalance on the prediction model. When compared to other data imputation and prediction models, our method achieves the best yield prediction result even when missing data is not pre-processed.

## 1 Introduction

Maize (*Zea mays* L.) is the largest grain crop in China, grown throughout the country. Accurate estimation of maize yield using environmental data and maize growth data before harvest is critical for food security and agricultural policy development. For example, accurate yield prediction helps growers and decision-makers adjust the scale of maize planting in a timely manner, as well as the government adjust agricultural policies in a timely manner to cope with the constantly changing maize market (Kang et al., 2020). The factors affecting maize yield are extremely complex. Temperature, precipitation, soil, humidity, and other meteorological factors, as well as their interactions, have a significant impact on maize yield (Ortiz-Bobea et al., 2018). Furthermore, the traits during growth (plant height, ear length, ear height, kernel numbers per row, etc.) can primarily reflect the maize yield (Liu and Basso, 2020).

The ability to accurately predict crop yields has been a challenge in agricultural production. Remote sensing, machine learning (ML) algorithms, and other technical methods have been widely used to predict crop yields. You et al. (2017) addressed the issue of insufficient training data in remote sensing images by using a dimensionality reduction technique to convert remote sensing images into pixel histograms, which were then trained using convolutional neural networks and long short-term memory models to predict county-level soybean yields in the United States. Zhu et al. (2021) synthetically used agrometeorological indicators and remote sensing vegetation parameters to estimate maize yield in the Jilin and Liaoning provinces of China. Ruan et al. (2022) combined remote sensing and climate data to develop a seasonal yield prediction model for wheat in the field. Eleven statistical and ML regression algorithms were adopted for regression prediction. The models provide a good idea for using multiple sources of data to predict yield. The implementation of crop yield prediction based on remote sensing necessitates a high spatial resolution of remote sensing images, which is easily influenced by weather and the professional level of data collectors. Nevertheless, ML-based models were used to predict crop yield, significantly improving model prediction and generalization performance. Kang et al. (2020) used a variety of ML algorithms (Lasso, Support Vector Regressor, Random Forest, XGBoost, Long Short-Term Memory, and Convolutional Neural Network) to predict county-level maize yields in 12 Midwest states. Their results demonstrate that seasonal crop yield forecasting benefits from both advanced algorithms and a diverse set of information about crop canopy, environmental stress, phenology, and soil properties. Ma et al. (2021a) developed a county-level corn yield prediction model using a Bayesian Neural Network to estimate yield and prediction errors. This model could not only accurately estimate the corn yield in normal years, but also accurately evaluate the corn yield in abnormal years with extreme weather. Khaki and Wang (2019) used a multi-layer perceptron based on soil and weather data to predict maize hybrid yields in the United States. Their results indicated that environmental factors had a greater effect on crop yield than genotypes.

Although the above works have produced good predictions, they all train and test models using environmental and crop data from a specific area (county or state). Due to significant differences in climate, soil, and other natural environments between regions, these models are difficult to use to predict crop yields in other regions. ML models developed within a specific spatial domain often lose validity when applied to new regions Ma et al. (2021b). Ma et al. (2021b) developed an unsupervised adaptive domain adversarial neural network. The model mitigated the impact of domain shift by projecting data from different domains into the same subspace, ensuring that the model could learn domain-invariant features while also performing accurate yield prediction. Although this model provides a novel approach to improving model transferability in crop yield prediction. However, neither the spatial correlation between different planting locations nor the correlation between different traits was considered.

In fact, there is a strong spatial correlation between maize yields in different planting areas, and there is also a strong intrinsic correlation between maize traits. For instance, planting locations that are close geographically have similar meteorological characteristics and grow similar maize varieties. Therefore, if one county has a splendid maize harvest within a specified year, its neighboring counties may also have a high maize yield (Fan et al., 2022). Furthermore, lodging rate is closely related to stalk thickness, cold tolerance is closely related to leaf color, and the interaction of these traits affects maize yield. Fan et al. (2022) used meteorological and soil data to construct a novel graph-based recurrent neural network that predicted maize yields in 2000 counties across 41 states in the United States. For the first time, this method used a graph neural network (GNN) to establish spatial correlation between different regions, resulting in accurate large-scale yield prediction. However, it did not consider the effect of the correlation between different maize traits on yield.

The quality of the trait data will also affect the yield prediction results. Unprocessed maize trait data collected at each maize planting location typically contains two types of problems: missing data and imbalanced data. The loss of data during field trials is a prevalent issue, often attributed to non-human factors such as natural disasters, including heavy rainfall. These events can lead to the toppling over of maize plants and subsequent data loss during the late growth period. Additionally, other factors may contribute to this problem, such as inadequate seed supply at specific trial sites, compromised seed germination rates, and errors made by data collectors (Yang et al., 2023). The sample imbalance is primarily caused by differences in planting scales across regions, as well as different maize growth data recording rules. For the problem of missing data, deleting missing values directly introduces bias in analysis, especially when the missing data is not randomly distributed, which may exacerbate the sample imbalance (Emmanuel et al., 2021). The incorrect imputation of missing values introduces noise and further reduces the accuracy of yield forecasting. In recent years, many general data imputation models have been used to solve the data missing problem, such as the multiple imputation chain equation (Xu and Qiu, 2022), self-attention graph convolution residual network (Zhang et al., 2022), low-rank matrix factorization method (Wang et al., 2019), generative adversarial network (Yoon et al., 2018), bipartite graph neural network (You et al., 2020), etc.

According to the investigation, there is a natural spatial correlation between meteorological characteristics, maize traits, and maize yield across neighboring planting locations (Fan et al., 2022). Furthermore, there are correlations between meteorological features and maize traits within the planting locations (Wang, 2001). These correlations provide important information for missing maize planting data imputation and yield prediction. Moreover, the yield can be regarded as a one-dimensional feature of the maize planting data; thus, the maize yield forecast can be regarded as the missing yield data imputation. Currently, the data imputation method based on a bipartite graph neural network achieves a better imputation effect by establishing associations between different types of features and data observation values (You et al., 2020). Inspired by this model, this paper proposes a maize yield prediction model based on a bipartite graph neural network. The model takes meteorological features and maize traits (partial deletion) as inputs and returns imputation results and yields prediction results for the maize deletion character. The specific research objectives are as follows: 1) A maize yield prediction model based on a bipartite graph neural network is proposed. Based on a bipartite graph neural network, the model establishes correlations between different maize planting sample data, different meteorological features and traits, and different traits, which can be used to achieve missing trait data imputation and predict maize yield in planting locations with different environments. 2) A new loss function is developed based on the gradient balance mechanism (Li et al., 2019), which effectively mitigates the negative impact of sample imbalance on maize yield prediction results. 3) First, eight data imputation models are used to fill in the missing data. The proposed method is then compared to several ML and deep learning prediction models. The results demonstrate that the proposed method could accurately fill in missing maize trait data and had the highest prediction accuracy.

## 2 Materials and methods

### 2.1 Study area

**Figure 1:**
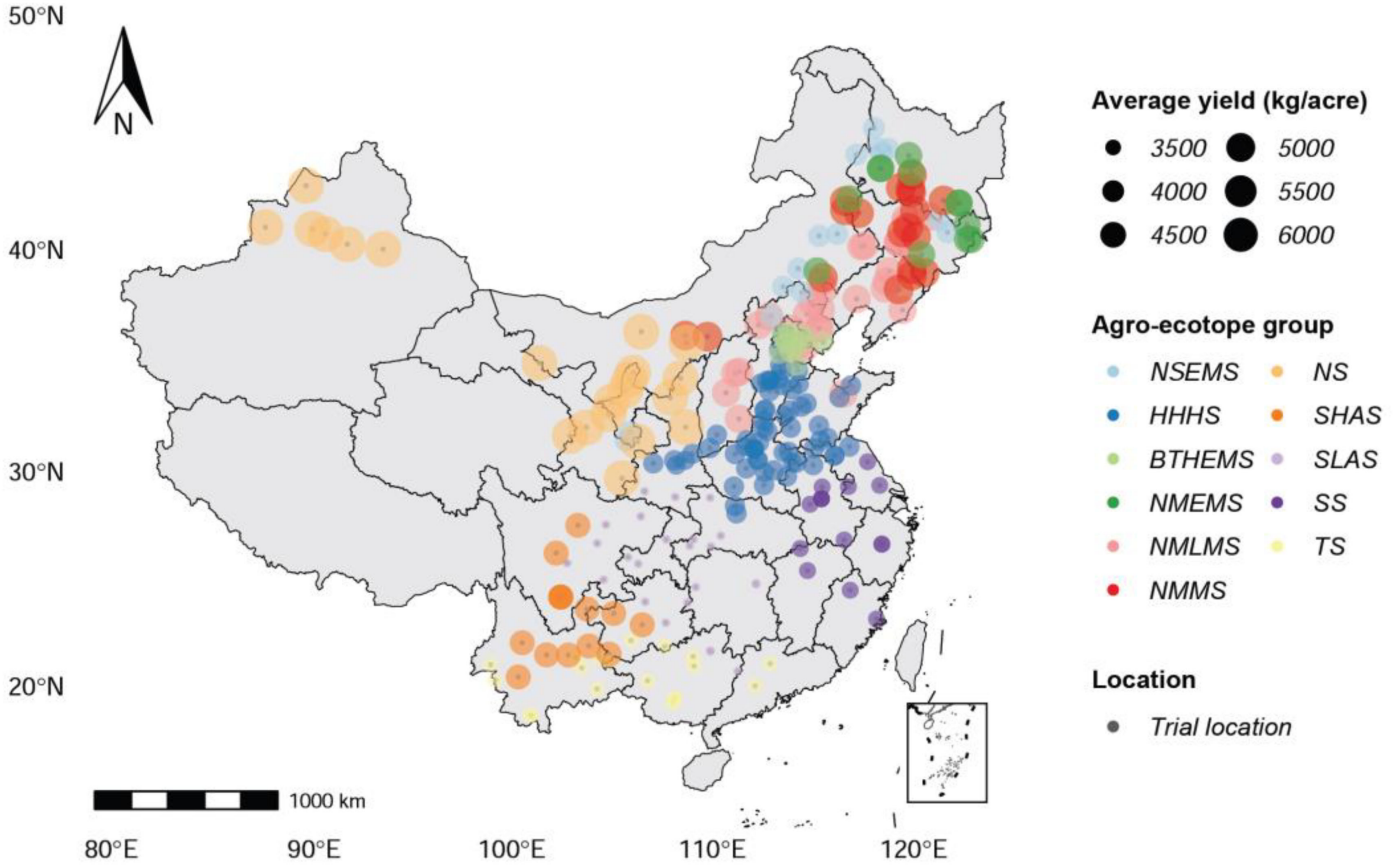
Map that shows the spatial distribution of 248 trial locations for 11 ecotopes in China in which maize is grown.

As shown in **Figure 1**, the study is focused on the primary area in which maize is grown in mainland China, which includes 11 ecotopes, such as Northern Super-Early-Maturity Spring Maize (NSEMS), Northeast and North China Medium-Maturity Spring Maize (NMMS), Northeast and North China Middle-Late-Maturity Spring Maize (NMLMS), Northeast and North China Middle-Early-Maturity Spring Maize (NMEMS), Southeast Spring Maize (SS), Huang-Huai-Hai Summer Maize (HHHS), Beijing-Tianjin-Hebei Early Maturity Summer Maize (BTHEMS), Tropical and Subtropical Maize (TS), Northwest Spring Maize (NS), Southwest Low Altitude Spring Maize (SLAS), and Southwest High Altitude Spring Maize (SHAS). Because of China’s vast land area and complex topography, maize planting regions are unevenly distributed throughout the country. There are significant differences in maize yield between different regions. The phenotype of maize varieties in multi-environment trials determines whether or not they can be certified and promoted. The trial data used in this study includes almost all of China’s major maize trial fields, totaling 248 trial locations.

### 2.2 Dataset

The maize Maize trial data from 248 trial locations across 11 ecotopes from 2017 to 2021, a total of 13,000 samples, included multidimensional maize trait feature values during growth and yield value at harvest. All the maize yields are measured in kg/acre, which is weighed gravimetrically after threshing. Each trait feature group has 20 dimensions, including planting location, planting date, maturity date, corn variety, grain color, corn cob type, stay-green, seedling leaf sheath color, axis color, anther color, ear rot resistance, big spot resistance, gray spot resistance, plant height, spike height, empty rod rate, spike length, bald tip length, row grain size, spike shank length, and spike thickness. There is some missing data in the maize trial data set, and the missing rate is approximately 18%.

Meteorological features from various maize planting locations are collected. All meteorological data are obtained from the China Meteorological Data Service Centre, which provides daily meteorological data for all of China’s counties. The downloaded useful meteorological data includes ten dimensions: daily maximum temperature, daily average temperature, daily minimum temperature, daily temperature difference, daily average ground pressure, daily average relative humidity, daily precipitation, daily average wind speed, daily maximum wind speed, daily wind rating, and daily sunshine hours. The growth cycle of the corn begins at the planting date and ends at the maturity date. To obtain the meteorological features associated with each group of corn, we extract meteorological data for each day in the corresponding county during the growth cycle and then calculate the mean and the variance of each group. The mean value describes the average level of each meteorological feature, while the variance describes the daily variation in each meteorological feature throughout the corn growth cycle. After this calculation, the original 10-dimensional meteorological features are transformed to 20 dimensions.

### 2.3 Data pre-processing

The meteorological and trait features in each dimension must be standardized before being fed into the Bipartite Graph Neural Network for data imputation and yield prediction. Because several maize traits are recorded in text format, to make subsequent calculations easier, we convert them into numerical labels. The text data included maize variety, seedling leaf sheath color, grain color, axis color, and anther color. For example, axis color labels can be white, red, pink, or purple, which corresponds to 1, 2, 3, or 4 in this article. Furthermore, there are significant differences between feature values in different dimensions. For instance, the value range of precipitation variance is [43.6, 739.7], whereas the value range of resistance to big spot is [0, 8]. The difference in dimensions tends to interfere with the rate of gradient descent of the proposed network, whereas standardization can reduce the impact of significant data differences on the model. Each dimension feature is processed using z-score normalization, as shown in Equation 1.


(1) $$t^{\prime }=\frac{t-\mu }{\sigma }$$

Where t represents raw data, μ denotes the mean, and σ represents the standard deviation.

### 2.4 Bipartite graph construction

**Figure 2:**
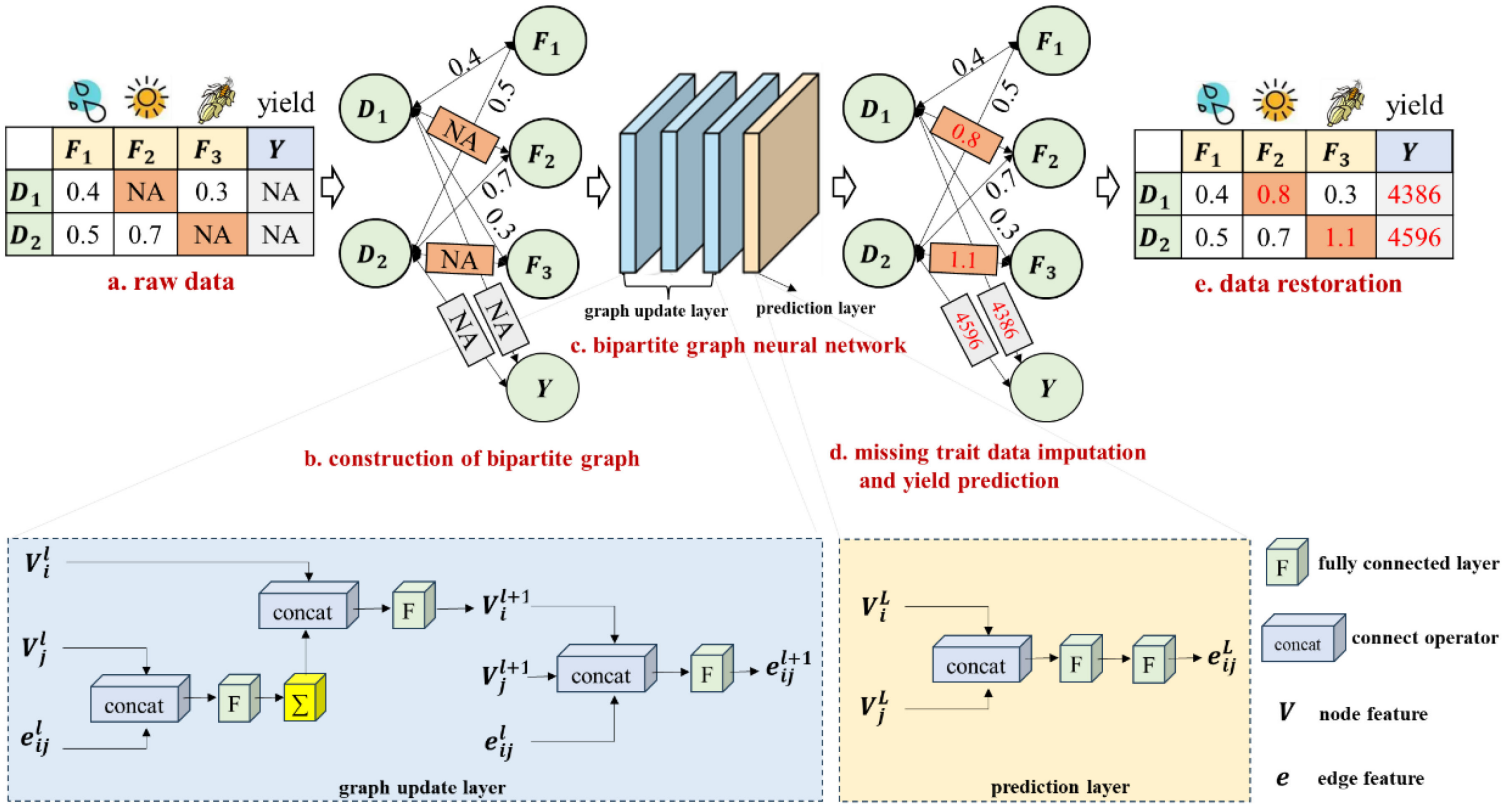
The overall structure diagram of the method. NA represents missing data; D denotes the observation item of maize planting; F indicates the trait feature item; Y signifies the yield.

The current superior data imputation method, which utilizes a bipartite graph neural network, has demonstrated superior performance in imputing missing values by establishing associations between diverse feature types and observed data values (You et al., 2020). Motivated by this approach, A maize yield prediction model based on a bipartite graph neural network is proposed. The overall structure diagram of the model is shown in **Figure 2**. First, the features (meteorological features and maize traits) with missing values are converted into a bipartite graph data structure (**Figures 2A, B**), with the missing values indicated by orange-red entries in the raw data table. Then, a bipartite graph neural network (**Figure 2C**) with three graph update layers and one prediction layer is constructed to output the results of missing trait data imputation and yield prediction (**Figures 2D, E**).

A bipartite graph is a special graph data structure in which nodes are divided into two types and edges are used to establish the relationship between the two types of nodes. Bipartite graph neural networks can learn potential correlations between each type of node. Therefore, the maize planting observation items D and the features F are considered two types of nodes in this graph, with the observed values serving as weighted edges between the observation item and feature nodes. The correlations between different maize planting data and the relationships among various features can be simultaneously explored using a bipartite graph neural network, thereby enhancing the accuracy of missing trait data imputation and maize yield prediction.

**Figure 3:**
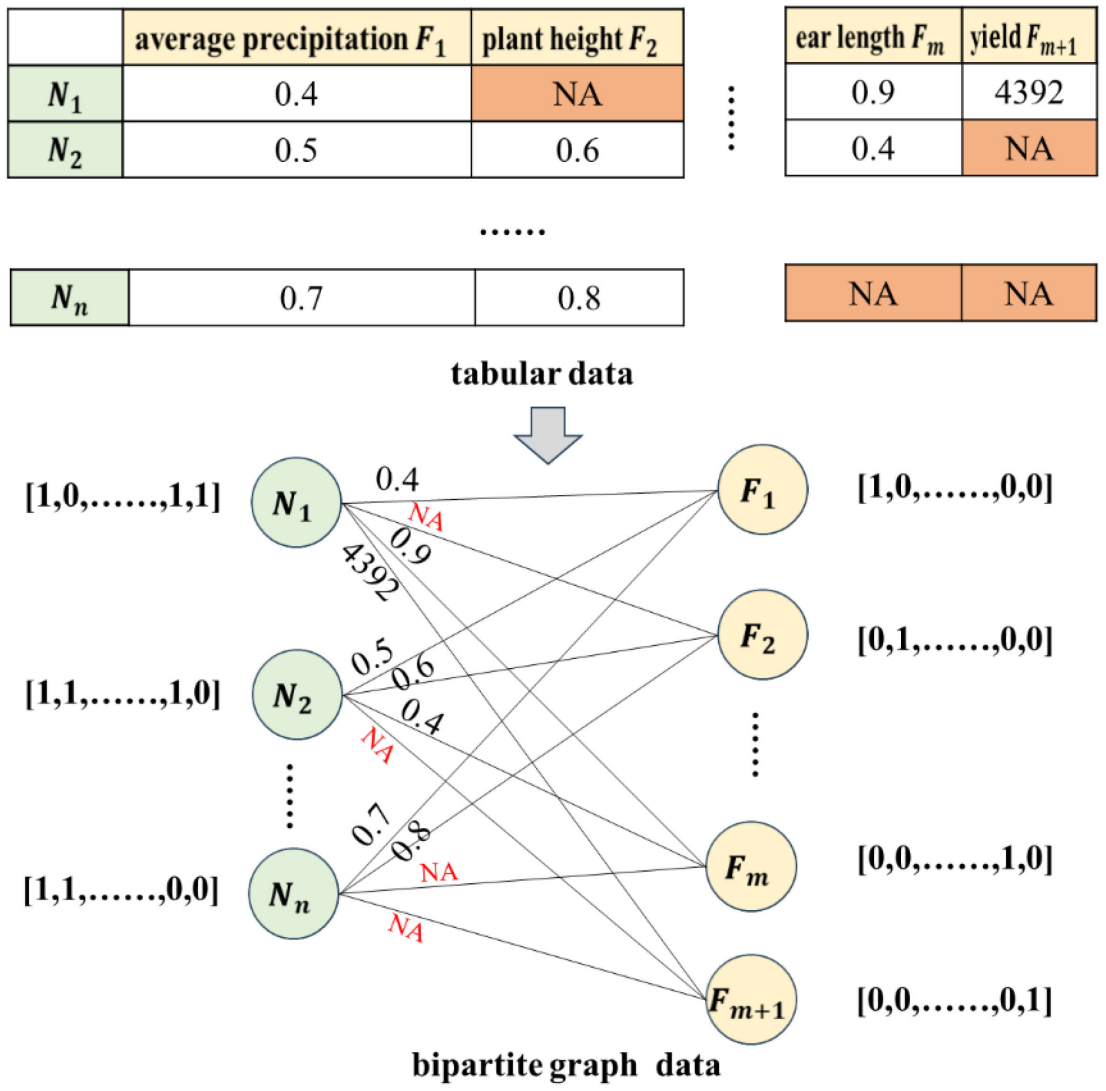
Bipartite graph construction process. NA represents missing data.

**Figure 3** shows the construction process of a bipartite graph. The first row (meteorological features, maize traits, and yield) and first column (observation item numbers) of the raw data table are treated as two types of nodes in the bipartite graph. The data values in the table are viewed as weighted edges on the graph. As shown in **Figure 3**, meteorological features and maize traits features have m dimensions, and n planting samples data are recorded in the table. All observation items numbers are used as a type of node in the bipartite graph, represented by $N_{i}$ , i∈(1,n) All the features (meteorological features, maize traits, and yield) are used as another type of node in the bipartite graph, represented by $F_{j}$ , j∈(1,m+1) . The table contains a total of n×(m+1) data values, so the bipartite graph contains a total of n×(m+1) edges. The $j^{th}$ feature of the $i^{th}$ set of data is represented by edge $e_{ij}$ . The data value is the weight of $e_{ij}$ , and if this value is empty, $e_{ij}=0$ .

The operation described above initializes the weight of each edge in the bipartite graph. However, the nodes in the bipartite graph only have physical meanings and lack real numerical representations, and different nodes in the graph represent completely different semantics. For example, meteorological features (such as precipitation, sunshine duration, etc.) and maize traits (such as plant height, ear height, etc.) belong to the same type of node, whereas the number of all planting samples (observation items) belongs to a different type of node. Therefore, it is crucial to use appropriate assignment methods to express each node’s semantic information when predicting yield. The value of each node was initialized using one-hot encoding, which used the simple and efficient 0 and 1 encoding to distinguish nodes. Each node is encoded as an m+1 dimensional 0 and 1 vector. The $j^{th}$ dimension feature of node $F_{j}$ is 1, and the features of the other dimensions are 0. The encoding of node $N_{i}$ depends on the feature values missing condition. If all feature values and real yield of the $i^{th}$ planting sample are recorded in the raw table, all features of node $N_{i}$ are 1. If the $j^{th}$ dimensional feature value of $i^{th}$ planting sample is missing, the $j^{th}$ dimensional feature of the node $N_{i}$ is 0. As shown in **Figure 3**, only the second-dimensional feature (plant height) of the first planting sample is missing. Therefore, the second-dimensional feature of node $N_{1}$ is 0, and the other features are 1. The initialized bipartite graph accurately preserved all key data in the original table by weighting the edges, and establishing an initial association between maize planting data as well as between maize features, laying the foundation for missing feature value imputation using the bipartite graph neural network and maize yield prediction.

### 2.5 Missing trait data imputation and maize yield prediction based on bipartite graph neural network

In this article, a bipartite graph neural network was designed for missing trait data imputation and maize yield prediction simultaneously, allowing for more accurate learning of the potential correlation within each type of node. This correlation can be mapped to two objective laws in the maize planting situation. First, there is a correlation between meteorological features and maize traits, which can also jointly affect maize yield (e.g., the correlation of nodes $F_{1}$ to $F_{m+1}$ in **Figure 3**). Second, there is a spatial correlation between meteorological features, maize traits, and yield between different planting samples data (e.g., the correlation of nodes $N_{1}$ to $N_{n}$ in **Figure 3**). Therefore, it has high applicability for missing trait data imputation and maize yield prediction using a bipartite graph neural network.

The bipartite graph neural network proposed in this paper consists of three graph update layers (blue rectangle in **Figure 2**) and one prediction layer (orange-red rectangle in **Figure 2**). The weights of the edges in the bipartite graph represent all of the data values in the table, which are crucial for maize yield prediction. Thus, using the weights of initial edges to impute missing feature values and predict maize yield is critical. In this study, the problem is solved using the graph update layer. Each graph update layer consists of two steps, the first of which uses edge embedding to update node features, as shown in Equation 2.


(2) $$V_{i}^{l+1}=L_{B}\left(Con\left[\sum_{j\in \varphi_{i}}L_{A}\left(Con\left[V_{j}^{l}\;,\;e_{ij}^{l}\right]\right)\;,V_{i}^{l}\;\right]\right)$$

As described in Section 2.4, the one-hot encoding is used to distinguish two types of nodes in bipartite graphs, and in the graph update layer, the update strategies of the two types of nodes are the same. Therefore, these two types of nodes are collectively referred to as V . In Equation 2, $V_{i}^{l}\;$ represents the $i^{th}$ target node in the $l^{th}$ feature update layer. Both $L_{A}$ and $L_{B}$ indicate the full connection layer. Con indicates a concatenation operation. $\varphi_{i}$ represents the set of all nodes connected by edges to the $i^{th}$ node, j represents the $j^{th}$ node between them, and $e_{ij}^{l}$ identifies the weight of the edge connecting node $V_{i}^{l}$ and node $V_{j}^{l}$ . Equation 2 shows the process of updating node features based on edge embedding in the $l^{th}$ graph update layer.

The second step of the graph update layer is to update the weights of edges based on node features. The process is as follows:


(3) $$e_{ij}^{l+1}=Con\left[V_{i}^{l+1},\;V_{j}^{l+1},\;e_{ij}^{l}\right]$$

The process fills a new feature vector for missing data values based on the node features and the weight of the previous layer’s edge. The design of the three-layer graph update layer allows the bipartite graph structure to fully learn high-order correlations between data. Finally, a prediction layer is constructed with node features obtained from the third layer of the graph update layer serving as inputs. The missing trait data imputation and yield prediction are accomplished using two fully connected layers (the orange-red rectangle in **Figure 2**). The dropout prevents the network from overfitting. For each layer of the graph update layer, the dropout hyperparameter is set to 0.1, indicating that each neuron in the graph update layer is discarded at random with a probability of 0.1.

### 2.6 Loss function

Because of the differences in planting scales between maize planting locations, the problem of sample imbalance often appears in the data. For example, maize planting in the northeast of China is relatively intensive, whereas there are fewer maize planting locations in the northwest, resulting in fewer samples of maize planting data collected in that region. Sample imbalances can easily cause overfitting of the prediction model, reducing prediction accuracy. The Gradient Harmonized Mechanism (GHM) is an effective approach to dealing with such data issues (Li et al., 2019). The influence of sample quantity differences on model training can be represented by a gradient, while the balance gradient reduces the negative influence of sample problems on the model. Currently, the GHM has shown promising results in areas such as object detection (Zhu et al., 2019; Wu et al., 2020) and remote sensing image segmentation (Zheng et al., 2020). The GHM is embedded to optimize $L_{1}$ loss function for solving regression problems, and the loss function $L_{GHM}$ in this paper is obtained, as shown in Equation 4.


(4) $$L_{GHM}=\frac{1}{N}\sum_{i=1}^{N}\left(|y_{i}-y_{i}^{t}\right|+\frac{y_{i}-y_{i}^{t}}{\sqrt{{\left(y_{i}-y_{i}^{t}\right)}^{2}+\alpha^{2}}})$$

Where N represents the number of training samples, $y_{i}$ represents the estimated yield, $y_{i}^{t}$ represents the true value of the yield, $\left|y_{i}-y_{i}^{t}\right|$ indicates the $L_{1}$ loss, $\frac{y_{i}-y_{i}^{t}}{\sqrt{{\left(y_{i}-y_{i}^{t}\right)}^{2}+\alpha^{2}}}$ indicates gradient harmonized mechanism, $\alpha^{2}$ is a constant, and the α value is set to 0.3 in this study. In general, the difference between estimated and actual yield values is greater at planting locations with less sample data. The mechanism dynamically adjusts the sample weight based on the difference between the estimated and true values to reduce the model’s interference from data imbalance.

### 2.7 Assessment of the model performance

The mean absolute error (MAE), root mean square error (RMSE), and coefficient of determination (R^2^) are used to assess the accuracy of yield prediction. R^2^ is the ratio of residual squares to total squares; the higher the value, the better the fit between predicted and actual yields. RMSE and MAE are used to calculate the degree of deviation between predicted and actual yields; the lower the value, the higher the prediction accuracy of the model. These evaluation metrics are mathematically expressed as Equations 5-7.


(5) $$MAE=\frac{1}{n}\sum_{i=1}^{n}{\left|\hat{y_{i}}-y_{i}\right|}^{2}$$

(6) $$RMSE=\sqrt{\frac{1}{n}\sum_{i=1}^{n}{\left(\hat{y_{i}}-y_{i}\right)}^{2}}$$

(7) $$R^{2}=1-\frac{\sum_{i=1}^{n}{\left(\hat{y_{i}}-y_{i}\right)}^{2}}{\sum_{i=1}^{n}{\left(\bar{y_{i}}-y_{i}\right)}^{2}}$$

Where n represents the number of samples, and in this study n = 13,000; $\hat{y_{i}}$ represents the predicted yield of the $i^{th}$ sample, and $y_{i}$ represents the true yield of the $i^{th}$ sample.

All of the experiments in this study are carried out on Ubuntu 18.04 with CUDA 10.0 and an NVIDIA Tesla P100 16 GB graphics card. The Pytorch library is used to develop and test the proposed model. The DGL library is used to build a bipartite graph neural network.

## 3 Results

### 3.1 Missing data imputation results comparison and analysis

In this study, there are 13,000 maize planting sample data, of which 7651 have no missing data. The effectiveness of the proposed bipartite graph neural network is evaluated in missing trait data imputation by randomly deleting values from 7651 planting sample data. The MAE between the data imputation result and the actual value serves as an indicator for evaluating the data imputation effect. Because the true missing rate of all data in the experiment is approximately 18%, we perform random missing of non-missing data in proportions of 10%, 20%, and 30% to verify the prediction accuracy of the data imputation algorithm and compare it to eight commonly used data imputation methods. The study compares eight data imputation methods: mean imputation, median imputation, chain imputation, K-nearest neighbor imputation, Singular Value Decomposition (SVD) model, GAIN model (Yoon et al., 2018), GraphRNA model (Huang et al., 2019), and SAT model (Chen et al., 2020). Mean imputation and median imputation are the simplest two types of data imputation methods. They deal with all missing values for each one-dimensional feature using the same data without considering sample differences, which can easily introduce data noise and reduce prediction accuracy. Chain imputation is a more robust imputation method than others because it employs the Monte Carlo method to deal with missing data and accurately estimates the posterior distribution of each dimensional feature. The core idea is that each missing value is modeled based on the observed non-missing value. K-nearest neighbor imputation and the SVD model are both effective data imputation methods based on ML. K-nearest neighbor imputation estimates missing values by leveraging data correlation across multiple dimensions. The SVD model achieves the matrix using iterative low-rank singular value decomposition and then estimates the missing data. In recent years, the GAIN, GraphRNA, and SAT models have all proven to be excellent deep learning methods. The GAIN model is based on generative adversarial networks, and the idea is that the generator computes the generated data using the actual observed real data, while the discriminator focuses on distinguishing between the real and generated data. The GraphRNA model incorporates a collaborative walking mechanism - AttriWalk - into the graph recursive network to learn node embedding, improve the ability to learn the representation of node features, and fill in missing node information using the graph node update process. The SAT model establishes a shared-latent space assumption for the attributes and structure of the graph to predict missing node attributes.

**Figure 4:**
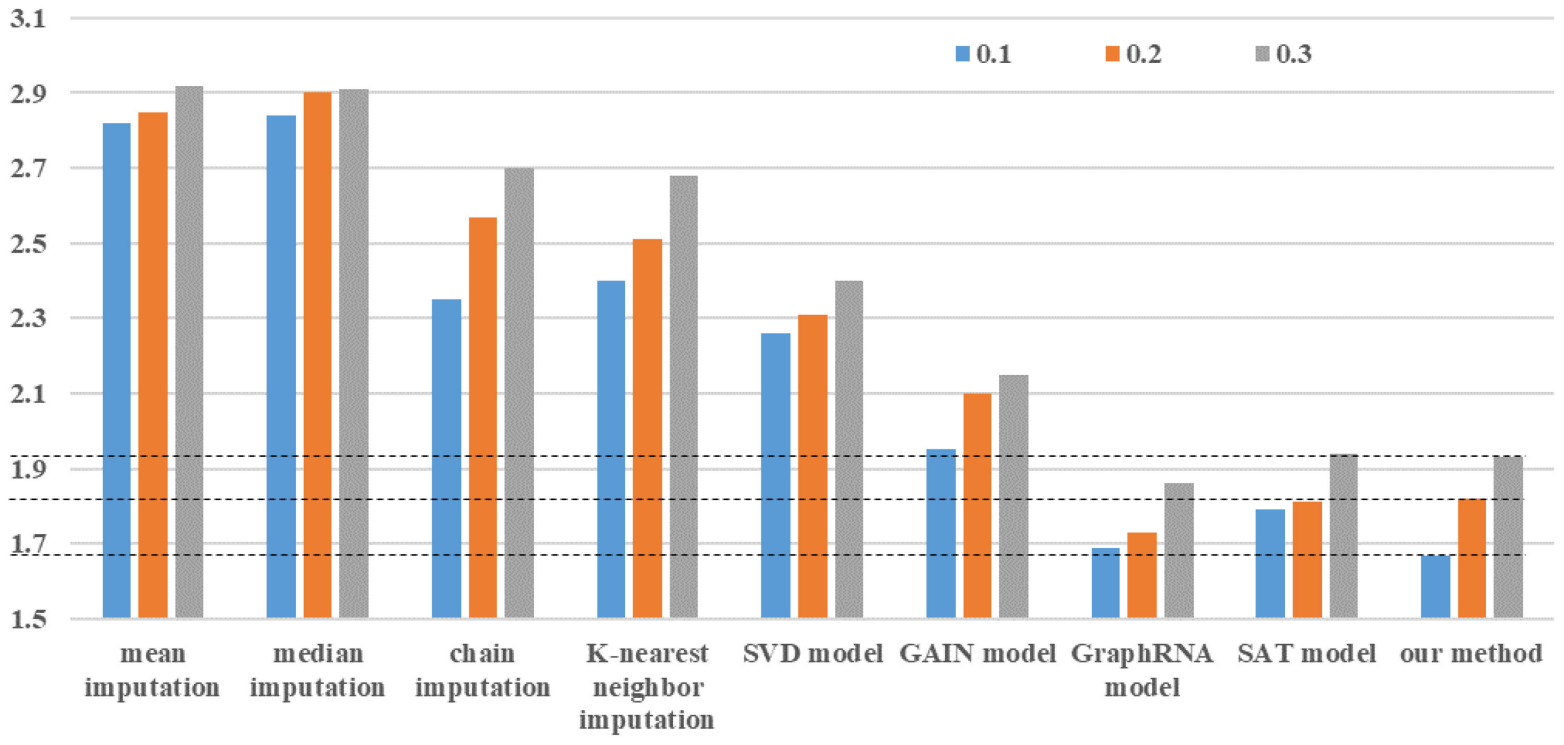
Mean absolute error of different data imputation methods with different data missing rates.

The method proposed in this article can also be used separately for missing maize trait features imputation, i.e., when constructing a bipartite graph neural network, only maize trait data is used and yield data is ignored. **Figure 4** shows a comparison of data imputation effects between our method and the other eight methods using the same data and missing rate. It is obvious that mean and median imputation have the worst prediction effect, and their imputation accuracy is basically not affected by the data missing rate. The GAIN model outperformed the three data imputation algorithms of chain imputation, K-nearest neighbor imputation, and the SVD model in terms of imputation accuracy, demonstrating the effectiveness of generative adversarial networks. The data imputation effects of the SAT model, the GraphRNA model, and our method are all at a relatively optimal level. When the data missing rate is 0.1, our method’s imputation effect is the best. When data missing rates are between 0.2 and 0.3, the GraphRNA algorithm produces the best data imputation results. These results demonstrate the robustness of using graph neural networks to impute missing maize traits, as the graph structure accurately expresses the potential association between different maize planting data. However, the above eight methods are limited to data imputation and lack the ability to perform both data imputation and prediction.

### 3.2 Maize yield prediction results comparison and analysis

The 13,000 planting sample data are divided into training and testing sets based on the number of missing features in each sample data. The training set contains 9,000 data samples with fewer than two missing features per sample, whereas the testing set contains 4,000 data samples with three or more missing features per sample. Compared to random partitioning, the data partitioning method used in this study not only reduces the interference of missing values on model training, but also visually displays the data imputation effect based on the test set.

This study compares the yield prediction model based on a bipartite graph neural network to eight other prediction models. Various models have been used, including Random Forest, adaptive enhancement (AdaBoost), gradient enhancement (GradientBoost), XGBoost model (Chen and Guestrin, 2016), TabNet model (Arik and Pfister, 2021), graph convolution network (GCN) (Kipf and Welling, 2016), graph attention network (GAT) (Veličković et al., 2017), and hierarchical graph representation learning (HGRL) (Ying et al., 2018). Random Forest, AdaBoost, and GradientBoost are built with functions from the Sklearn library. XGBoost has designed an extreme gradient-boosting algorithm that can solve classification and regression problems accurately and quickly. TabNet processes tabular data using sequential attention and has a high level of representation learning and interpretability. Based on the graph convolutional network, the graph attention network adds an attention mechanism to calculate the importance of the neighbor node to the target node by attention. The graph hierarchical representation network enhances the expression of the graph hierarchy using the graph convolutional network. However, because these prediction models lack data imputation capabilities, the GraphRNA model is used first to impute missing data, and the yield prediction is then achieved based on these methods. The algorithm in this study uses an end-to-end bipartite graph neural network to perform data imputation and yield prediction simultaneously.

**Figure 5:**
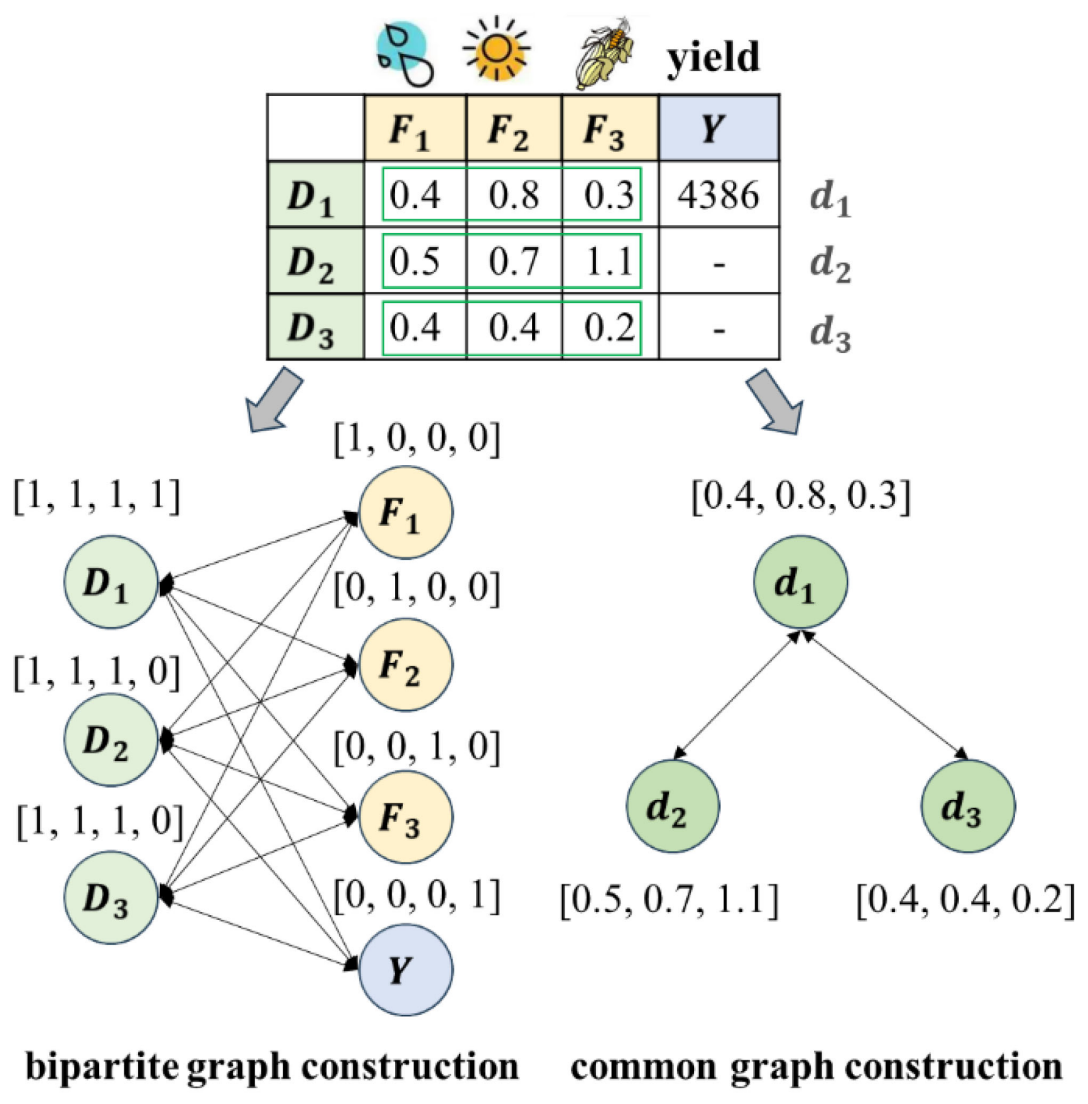
The comparison of bipartite graph construction process and graph construction process.

When using a graph neural network to predict maize yield, the tabular data is converted into a regular graph structure. Specifically, the 13,000 sample data of multidimensional maize features are converted into a graph structure. The graph structure consists of 13,000 nodes. The graph edges are used to connect nodes with high feature similarity, which is determined by calculating the Euclidean distance between the features of various data nodes. **Figure 5** compares the bipartite graph construction process and the graph construction process. To ensure fairness in comparison, the proposed method and the three neural network-based methods use the same training epochs (20000 epochs).

The evaluation results of the proposed model and eight other comparison models for predicting maize yield are presented in **Table 1**. The most effective method is highlighted in bold. It is clear that graph neural networks (GCN, GAT, and HGRL) outperform other methods in terms of prediction accuracy. This is mainly because of the graph neural network’s continuous aggregation and updating of nodes, which fully exploits the correlation between different maize planting sample data and significantly improves yield prediction accuracy. The method proposed in this paper does not require the data imputation algorithm to impute missing data first and is significantly superior to all other methods that perform data imputation and yield prediction separately in RMSE and MAE indicators. The main reason is that yield prediction using a bipartite graph neural network considers the correlation between different meteorological features and maize traits. Moreover, the model proposed in this study is end-to-end, providing significantly better convenience and training time than other methods for missing data imputation and yield prediction.

**Table 1:** The performance of different models in predicting maize yield under different data imputation methods.

Prediction method	RMSE	MAE	$R^{2}$
Random Forest	77.26	62.11	0.607
AdaBoost	78.35	61.96	0.694
GradientBoost	87.52	69.30	0.864
XGBoost	76.45	60.23	0.727
TabNet	65.70	51.17	0.688
GCN	52.53	36.15	0.864
GAT	51.34	35.43	0.884
HGRL	49.69	34.79	0.875
Our method	**46.28**	**33.18**	**0.893**

The most effective method is highlighted in bold; Underlined text indicates the superiority of graph neural networks (GCN, GAT, and HGRL) in terms of prediction accuracy.

**Figure 6:**
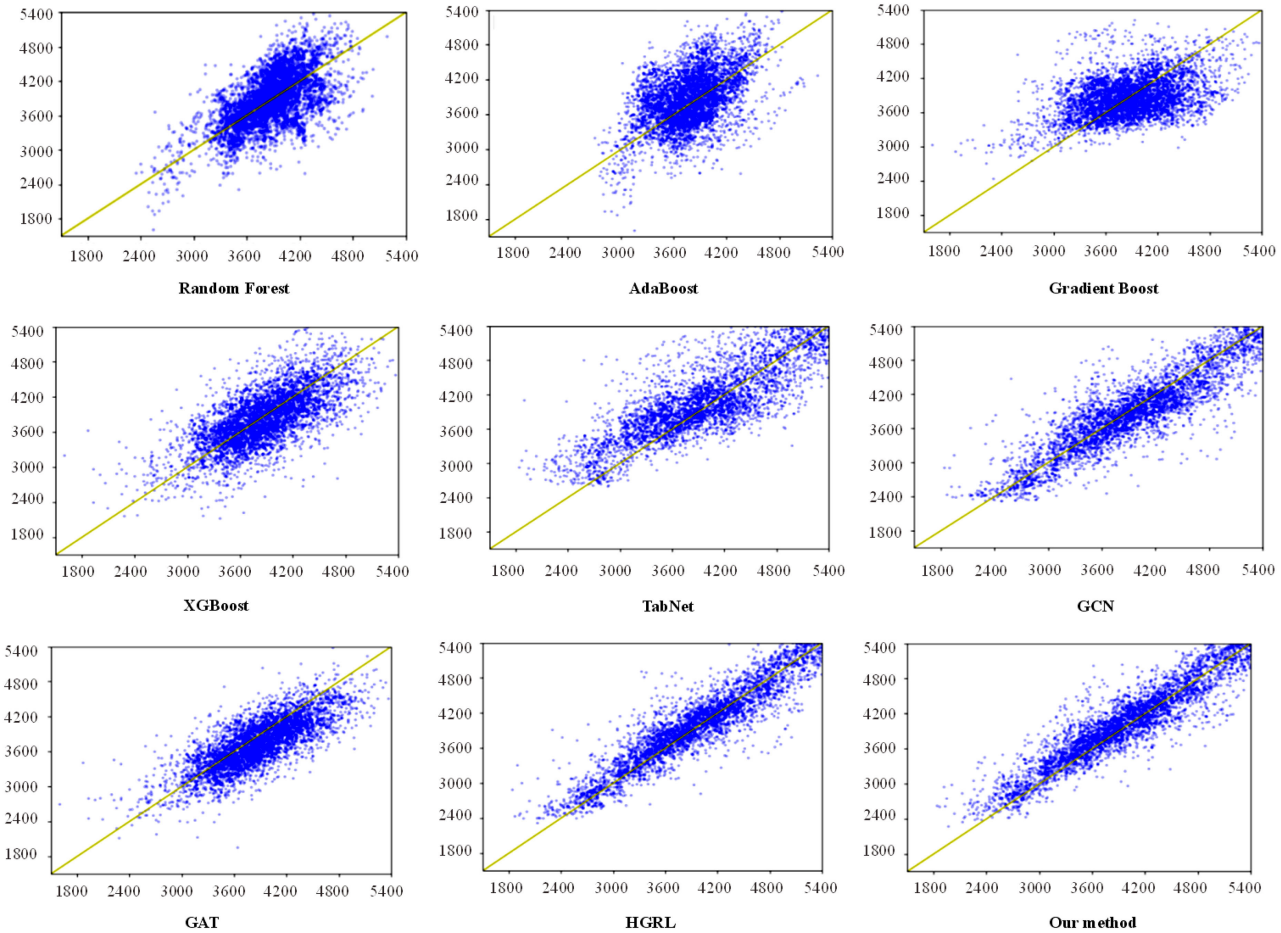
The scatter plots between predicted yield and true yield.

**Figure 6** depicts a scatter plot of the yield prediction results from the other eight prediction methods and the method proposed in this study. The horizontal axis represents actual production, while the vertical axis represents predicted production. The yellow line indicates that the predicted yield is equal to the actual yield. The greater the number of blue dots fitted to the yellow line, the better the prediction effect of the model. The number of blue dots equals the number of test sets. In this study, there are 4000 planting samples of test data, so there are 4000 blue dots in each subgraph of **Figure 6**. These graphs demonstrate that the scatter plots for Random Forest, AdaBoost, Gradient Boost, XGBoost, TabNet, and GCN methods are more scattered, resulting in a poor prediction effect. GAT cannot accurately predict samples with extremely high and low yields. This is mainly because the GAT network assigns low weight to data with an abnormal yield. The prediction result of HGRL is similar to the method proposed in this study, but HGRL only has prediction capabilities, and maize planting data must be pre-imputed before using this model. Furthermore, in the same experimental environment and number of training epochs, GAT takes 6.9 hours to train the model, while HGRL takes 7.5 hours, and our method takes about 4 hours.

### 3.3 Ablation experiment

The best yield prediction results are obtained when the graph update layer is three layers, random inactivation is used, and the hyperparameter of $L_{GHM}$ loss is set to 0.3. Three groups of ablation experiments are carried out to verify the effects of random inactivation, the number of update layers in the graph, and the loss function setting on the maize yield prediction. **Table 2** shows the specific results of the ablation experiment. The first part of **Table 2** verifies the effect of random inactivation on yield prediction. Random inactivation only affects the graph update layer, and neurons in each layer are discarded at a probability of 0.1. The comparison reveals that random inactivation significantly enhanced the effect of yield prediction. ( RMSE and MAE decreased by 4.04 and 2.25, respectively, and $R^{2}$ increased by 0.044).

**Table 2:** The main ablation results in three parts.

Items	RMSE	MAE	$R^{2}$
Random inactivation is not used	50.32	35.43	0.849
Random inactivation is used	46.28	33.18	0.893
the number of graph update layers is 2	47.13	34.11	0.852
the number of graph update layers is 4	47.79	34.07	0.861
the number of graph update layers is 3	46.28	33.18	0.893
$L_{1}$ loss is used	48.92	35.17	0.860
$L_{2}\;$ loss is used	50.06	36.14	0.853
$L_{GHM}$ loss (( α =0.5) is used	47.13	34.06	0.874
$L_{GHM}$ loss ( α =0.7) is used	47.06	34.11	0.882
$L_{GHM}$ loss ( α =0.3) is used	46.28	33.18	0.893

The second part of **Table 2** examines the impact of the number of graph update layers on maize yield prediction results. The number of graph update layers increases, as does the number of network parameters and training time. When the number of network layers increases from two to three, the prediction accuracy improves significantly. However, when the number of graph update layers is increased to four, prediction accuracy decreases significantly. This could be because each graph update layer contains a large number of nonlinear changes, and each change is equivalent to losing a part of the original information about features, resulting in network degradation.

The third part of **Table 2** examines the effect of different loss function settings on maize yield predictions. $L_{1}$ loss and $L_{2}$ loss are commonly used loss functions to solve regression problems. However, compared to $L_{1}$ loss, $L_{2}$ loss is more sensitive to outliers, and it is more prone to occur that the model ignores other normal data to minimize outliers. Therefore, in the yield prediction model proposed in this study, $L_{1}$ loss is superior to $L_{2}$ loss. The $L_{GHM}$ loss proposed in this study is based on the $L_{1}$ loss and adds a GHM, which includes parameter α , to adjust the influence of sample imbalance on the model. It is proved that the yield prediction effect is best when α is 0.3.

## 4 Discussion

There is a strong spatial correlation between maize yields in different planting regions, as shown in **Figure 1**. Northern China has a higher average maize yield than southern China. Planting regions with similar geographical locations share similar meteorological features, maize varieties, and yields. Thus, it is necessary to incorporate geospatial and temporal knowledge into crop yield prediction while taking advantage of the spatial structure of the data. Related studies demonstrate that incorporating knowledge about a county’s geospatial neighborhood and recent historical data can significantly improve the prediction accuracy of deep learning methods for crop yield prediction, as opposed to previous approaches that assumed neighboring counties were independent samples (Fan et al., 2022; Yang et al., 2023). Climate variations affected maize traits and, as a result, grain yield, and there were correlations between maize traits (Li and Tao, 2023). However, previous studies have not yet considered the correlation between data features, such as the correlation between meteorological features and maize traits, or the correlation between different maize traits.

Furthermore, missing and unbalanced data negatively affect model prediction results. Real-world maize planting data suffers from a data unbalance problem, as planting scales vary unevenly in both spatial and data domains. This imbalance can easily lead to prediction methods that favor the side with more data. This study redesigned the loss function to effectively address the problem of data imbalance. Prediction model studies that use ML methods rarely discuss the presence and treatment of missing data. Although many types of ML methods include built-in capabilities for dealing with missing values, these strategies are rarely used. Instead, most ML-based prediction model studies use complete case analysis or mean imputation (Nijman et al., 2022). In the research on crop yield prediction model construction, strategies for missing data imputation and data set expansion have been proposed, such as yield data compensation methods and graph neural networks (Zhang et al., 2023; Yang et al., 2023). Nonetheless, the methods proposed in the preceding study are limited to data imputation and do not have the ability to perform both trait missing data imputation and yield prediction.

To solve the problems mentioned above, the maize yield prediction model proposed in this study uses a bipartite graph neural network. The model establishes the correlation between different maize planting sample data, between meteorological features and traits, and between different traits through a bipartite graph neural network. It can simultaneously impute missing trait data and predict maize yield at planting locations with different environments and achieve good yield prediction accuracy (**Table 1**). The experimental results demonstrate that, when compared to general ML and deep learning methods, graph neural network methods perform significantly better in maize yield prediction. The reason for this is that graph neural network-based methods can continuously aggregate and update nodes, allowing them to fully exploit the high-order spatio-temporal correlation between each set of maize data. The proposed bipartite graph-based neural network model outperforms the other three models (GCN, GAT, and HGRL), with an increase in $R^{2}$ of 0.9% and decreases in RMSE and MAE of 3.41 and 1.61, respectively. This is due to the extra mining of correlations between meteorological features and maize traits. Compared to other methods that require imputed missing data and yield prediction, the end-to-end model presented in this study has better operation convenience and training time under the same experimental environment and number of training epochs.

## 5 Conclusions

To address the issue of missing maize trait data, this study proposes a maize yield prediction method based on a bipartite graph neural network. The maize planting sample data are first transformed into a bipartite graph data structure, and then a maize trait missing data imputation and yield prediction model based on a bipartite graph neural network is created. The model investigates high-order correlations among various maize planting sample data and the correlations among different features, increasing the accuracy of yield predictions. Furthermore, a loss function based on the GHM is used to effectively reduce the impact of sample imbalances between planting locations on model performance. The comparison results with various data imputation methods and prediction models demonstrate that the end-to-end model proposed in this paper achieves optimal yield prediction results without the need for additional data imputation. In the future, this study will improve the bipartite graph neural network using the attention mechanism to assess the strength of the correlation between maize planting data. The model will be applied to other fields, such as biology, to solve the prediction problem in the presence of missing data.

## Data Availability

The original contributions presented in the study are included in the article/supplementary material. Further inquiries can be directed to the corresponding authors.
